# Modulation of obesity-induced inflammation by dietary fats: mechanisms and clinical evidence

**DOI:** 10.1186/1475-2891-13-12

**Published:** 2014-01-29

**Authors:** Kim-Tiu Teng, Chee-Yan Chang, Lin Faun Chang, Kalanithi Nesaretnam

**Affiliations:** 1 Product Development and Advisory Services, Malaysian Palm Oil Board (MPOB), 6 Persiaran Institusi, Bandar Baru Bangi, 43000 Kajang, Selangor, Malaysia; 2 Department of Molecular Medicine, Faculty of Medicine, University of Malaya, 50603 Kuala Lumpur, Malaysia; 3 Department of Medicine, Faculty of Medicine, University of Malaya, 50603 Kuala Lumpur, Malaysia

**Keywords:** Inflammation, Obesity, Dietary fats, Saturated fatty acids, Polyunsaturated fatty acids, Adipose tissue

## Abstract

Obesity plays a pivotal role in the development of low-grade inflammation. Dietary fatty acids are important modulators of inflammatory responses. Saturated fatty acids (SFA) and n-6 polyunsaturated fatty acids (PUFA) have been reported to exert pro-inflammatory effects. n-3 PUFA in particular, possess anti-inflammatory properties. Numerous clinical studies have been conducted over decades to investigate the impact of dietary fatty acids on inflammatory response in obese individuals, however the findings remained uncertain. High fat meals have been reported to increase pro-inflammatory responses, however there is limited evidence to support the role of individual dietary fatty acids in a postprandial state. Evidence in chronic studies is contradictory, the effects of individual dietary fatty acids deserves further attention. Weight loss rather than n-3 PUFA supplementation may play a more prominent role in alleviating low grade inflammation. In this context, the present review provides an update on the mechanistic insight and the influence of dietary fats on low grade inflammation, based on clinical evidence from acute and chronic clinical studies in obese and overweight individuals.

## Introduction

Obesity is a global epidemic in both developed and developing countries. In the United States alone, it is estimated that approximately 69% of adults are overweight (BMI ≥25 kg/m^2^) and 36% are obese (BMI ≥30 kg/m^2^) [1]. Obesity is associated with a cluster of metabolic syndrome, which is characterized by hyperglycemia, hypertension, dyslipidemia, reduced high density lipoproteins (HDL) cholesterol and abdominal obesity. Taken together, these metabolic disorders are closely linked to chronic inflammation. Numerous studies have reported that elevated adiposity is associated with increased plasma pro-inflammatory cytokines [2-4].

Weight loss is known to improve obesity-associated metabolic disorders, in particular low grade inflammation [5,6]. However, other dietary interventions aiming at improving obesity-induced inflammation have not been explored in detail. Long chain fatty acids are potent inflammatory mediators [7]. *In vivo* and *in vitro* studies have shown the pro-inflammatory effects of saturated fats (SFA) [8-10] and polyunsaturated fats (PUFA), in particular n*-*6 PUFA [11]. In contrast, n-3 PUFA, namely docosahexaenoic acid (DHA) and eicosapentaenoic acid (EPA) have anti-inflammatory properties [12-14]. Nevertheless, the evidence based on clinical studies in this respect is limited and controversial. In this context, the present review provides an update on the mechanistic aspects and the effect of dietary fats on low grade inflammation, based on clinical evidence from acute and chronic clinical studies in obese and overweight individuals.

## Obesity-induced inflammation

Numerous studies found that compared to healthy lean individuals, overweight and obese individuals have higher pro-inflammatory cytokines and lower anti-inflammatory cytokines [15,16]. Obesity is characterized by having a greater number of adipose tissue (hyperplasia) and an increase in the size of adipocytes (hypertrophy) [17,18]. The aforementioned conditions lead to oxygen depletion in adipose tissue hence causing adipocyte cell death. In addition, the excess storage of triacylglycerols (TAG) from dietary intake results in an excessive influx of free fatty acids into blood circulation. Taken together, this can lead to low-grade inflammation characterized by the over production of pro-inflammatory adipocytokines [19].

Adipocytes mediate inflammatory response by regulating the release of free fatty acids and adipocytokines, in particular tumor necrosis factor-α (TNF-α), interleukin-6 (IL-6), and monochemoattractant protein*-*1 (MCP-1) [17,20]. MCP-1 stimulates the recruitment of macrophages into adipose tissue via MCP-1/CC chemokine receptor 2 (CCR2) cascade [21,22]. The findings of macrophage infiltration in adipose tissue provide a mechanistic insight into the obesity-induced low-grade inflammation. This obesity-induced inflammatory state involves a phenotypic switch in adipose tissue macrophage from M2 to M1 state [23,24]. The detailed mechanism has been reviewed [25]. In brief, instead of converting monocytes to M2 macrophages, the migration of monocytes from the circulation to the macrophage clusters surrounding dead adipocytes switches M2 to the M1 state. M2 macrophages are also known as alternatively activated macrophages, producing anti-inflammatory cytokines such as interleukin*-*10 (IL-10) and interleukin*-*1Ra (IL-1Ra) [17,22,23,25]. M1 macrophages are defined as classically activated macrophages, and produce high concentrations of pro-inflammatory cytokines i.e. TNF-α and IL-6. This phenotypic switch of M2 to M1 is characterized by a reduction in IL-10 and arginase production, in conjunction with an increase in pro-inflammatory TNF-α production [23,26].

## Modulation of inflammation by dietary fatty acids: mechanistic insights

### Saturated fatty acids

**Figure 1:**
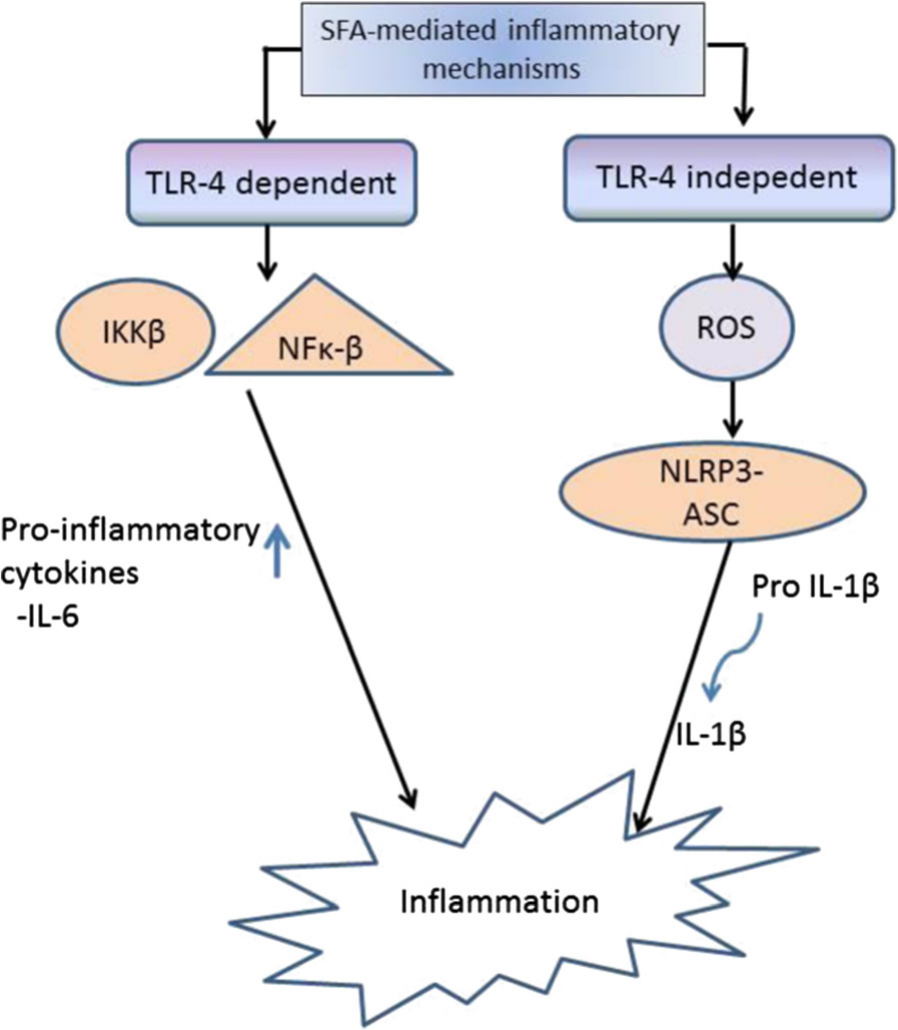
**TLR-4, toll-like-receptor 4; SFA, saturated fatty acids.** SFA stimulate inflammation through TLR-4 dependent and independent pathways.

Numerous *in vitro* and *in vivo* studies have shed light on the inflammatory effect of SFA [27-29]. Evidence from a substantial number of studies has reported that SFA stimulate inflammatory response by a pathway involving Toll-like receptors (TLR) (Figure 1). TLR are a class of pattern recognition receptors that play a crucial role in an innate immune system (reviewed in [30]). Elevated levels of TLR-4 have been reported in the obese state whereby the expression was found in many insulin target tissues such as liver, muscle, brain, adipose tissue, vasculature and pancreatic β-cells [31]. The absence of TLR in studies on mice demonstrated the diminished effect of insulin resistance modulated by pro-inflammatory pathways [32,33].

SFA is the acyl component of lipopolysaccharides (LPS), which is a ligand of TLR-4. It was reported that glycoproteins e.g. myeloid differentiation protein 2 (MD2) and a cluster of differentiation 14 (CD14) are needed for the binding of SFA to TLR-4 [34]. It has also been suggested that there are two pathways involved in the SFA-mediated inflammatory mechanisms, namely TLR-4-dependent and TLR-4-independent pathways (reviewed in [25]). SFA have been reported to induce TLR-4-dependent gene expression by promoting dimerization of TLR-4, a necessary step in receptor activation, within specialized lipid domains termed “lipid rafts” in macrophages [35]. The stimulatory effects of SFA, lauric, palmitic and stearic acids in particular, have been found to increase IL-6 gene expression in macrophage via the TLR-4 dependent pathway [36]. In line with this, stearic acid has also been found to stimulate the release of MCP-1 expression via TLR-4 [37]. TLR-4 activates pro-inflammatory pathways by stimulating the expression of transcription factors, including IKKβ and NF-κB [31,32].

Other than the TLR-dependent pathway, SFA stimulate pro-inflammatory mechanisms through the TLR-independent pathway by producing reactive oxygen species (ROS). ROS activate nucleotide-binding domain, leucine-rich repeat containing family, pyrin domain-containing 3 (NLRP3) inflammasome [38] that forms a complex with apoptotic speck protein (ASC), known as NLRP3- ASC inflammasome complex [39]. This complex regulates the cleavage of interleukin-1β (IL-1β) from pro-IL-1β [40,41]. The release of IL-1β decreases insulin signalling in insulin target cells, providing a possible SFA-mediated inflammatory response leading to insulin resistance [42,43].

### Polyunsaturated fatty acids

**Figure 2:**
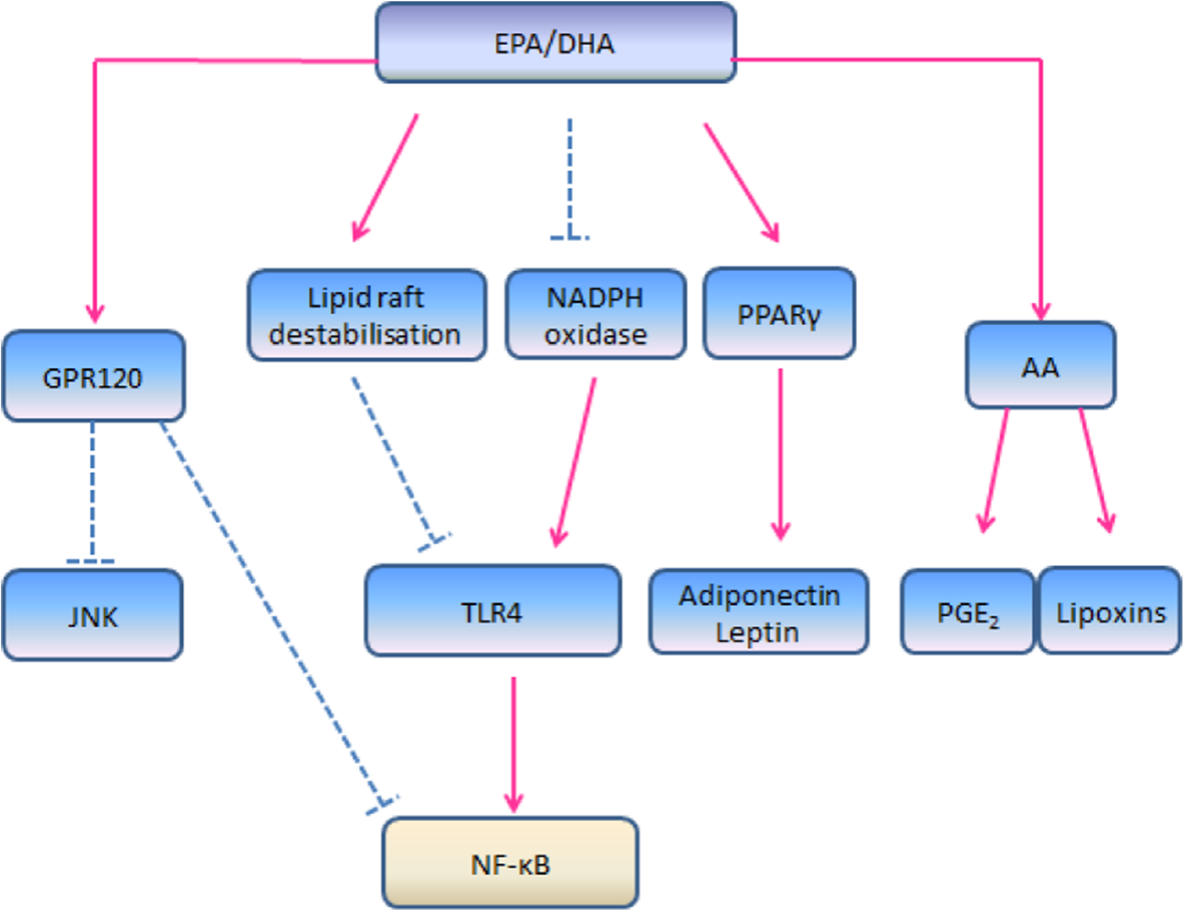
**Dotted line, inhibit; solid line, activate.** EPA, eicosapentaenoic acid; DHA, docosahexaenoic acid; GPR120, G protein-coupled receptor 120; JNK, JUN NH_2_-terminal kinase; TLR-4, toll-like-receptor 4; NF-κB, nuclear factor kappa-β; PPARγ, peroxisome proliferator-activated receptor γ; AA, arachidonic acid; PGE_2_, prostaglandins E2. Anti-inflammatory mechanisms of EPA and DHA. EPA and DHA inhibit NF-κB and JNK through binding with GPR120. Incorporation of these n-3 PUFA disrupts the translocation of TLR-4 into lipid raft, thus inactivates NF-κB pathway. Besides, EPA and DHA interfere with the TLR-4 signaling pathway via the downregulation of NADPH oxidase production, which results in the inhibition of NF-κB pathway. These fatty acids also activate PPARγ and, result in the upregulation of adiponectin and leptin secretion. In addition, the intake of EPA and DHA leads to antagonism of n-6 fatty acid arachidonic acid (AA).

n*-*6 PUFA, also known as linoleic acid (LA) is thought to be pro-inflammatory and it can be converted into arachidonic acid (AA) [44]. AA is the major substrate for eicosanoids production, which plays an important role in regulating inflammatory and immune responses [45]. Eicosanoids consist of prostaglandins, prostacyclins, thromboxanes, and leukotrienes. Biosynthesis of eicosanoid products are regulated by three major enzymes, namely cyclooxygenases, lipoxygenases and cytochrome P450s [46]. Most of the AA-derived eicosanoid products are pro-inflammatory, except prostaglandins E2 (PGE_2_) and lipoxins which have anti-inflammatory effects [47,48].

n*-*3 PUFA, namely linolenic acid (ALA) is known to exhibit anti-inflammatory properties [12,14,49]. Deep sea fish like salmon and mackerel are sources of n-3 PUFA. n*-*3 PUFA can be converted into EPA and DHA [44]. A number of reviews have been reported on the anti-inflammatory mechanisms of n-3 PUFA (Figure 2) [26,45,50]. One of the anti-inflammatory effects of n*-*3 PUFA is the downregulation of pro-inflammatory cytokines in adipose tissue such as TNF-α, IL-6 and MCP-1, which can be explained by several possible pathways [34,50-53]. The first possible pathway is the binding of EPA and DHA with the G protein*-*coupled receptor 120 (GPR120) [54]. Activated GPR120 internalizes as plasma membrane with β-arrestin2 to form GPR120/ β-arrestin2 complex. The complex which then associates with TAB1, results in the inhibition of TAK1 and prevents the downstream signalling to NF-κB and JNK systems [53].

While SFA were found to stimulate TLR-4 signalling, EPA and DHA were found to inhibit the TLR-4 signalling pathway [34]. The TLR-4 signalling pathway may be blocked by DHA and EPA through the downregulation of nicotinamide adenine dinucleotide phosphase-oxidase (NADPH oxidase) production [50]. NADPH oxidase induces ROS production, which is found to be necessary for TLR-4 signalling [51]. In addition, the incorporation of DHA into lipid membrane disrupts the translocation of TLR-4 into lipid raft, thus inhibiting the TLR-4 signalling pathway [50,55]. The disrupted TLR-4 signalling pathways lead to the inhibition of NF-κB thus resulting in the downregulation of inflammatory responses [53,54]. These findings indicate that the intake of EPA and DHA reduces the secretion of pro-inflammatory cytokines, and it may also be possible that these n-3 PUFA play a role in preventing macrophage infiltration into adipose tissue [50]. Findings from human [49,56] and rodent [57] studies reported that the production of adiponectin increases with a higher intake of EPA and DHA. However, the upregulation of adiponectin secretion may be associated with peroxisome proliferator- activated receptor γ (PPARγ) activation [57,58]. The upregulation of leptin by DHA is found to have a similar pathway with adiponectin [50]. It was suggested that a diet rich in EPA and DHA results in a higher incorporation of these fatty acids into phospholipid membrane, compared to AA. Therefore, a higher intake of EPA and DHA may reduce the production of AA-derived eicosanoid products, thereby providing another n-3 PUFA induced anti-inflammatory effect [47].

## Clinical evidence

Numerous prospective epidemiological studies have reported association between dietary fats and low grade inflammation. However, it is noteworthy that the effect may be speculative based on the association between changes in plasma fatty acids and levels of inflammatory cytokines. Clinical evidence provides a direct measurement of the impact of dietary intake on circulating inflammatory markers. Hence in the present review, clinical studies were identified by searching Pubmed, EMBASE, Cochrane databases. Inclusion criteria were English language articles reporting the measurement of at least one inflammatory marker including cytokines, vascular adhesion molecules, C-reactive proteins (CRP) in both postprandial and fasting measurements following the ingestion of high fat meals or dietary intervention (3-week and above) in overweight and obese individuals (BMI ≥ 25 kg/m^2^ or waist circumference > 80 cm for women and >90 cm for men) for the last 10 years. Studies investigating subjects under 18 y, lean and healthy, taking antioxidant supplements, pregnant and lactating, having chronic inflammatory related diseases were excluded. A total of 9 papers on acute studies and 12 papers on chronic studies were included in the following discussion.

## Acute dietary intake

### Amount of dietary fats on postprandial inflammatory response

Postprandial lipemia provokes the release of pro-inflammatory markers and impairs insulin response. A repetitive exposure of endothelial cells to pro-inflammatory cytokines leads to the progression of chronic diseases such as diabetes and cardiovascular diseases [59,60]. Previous studies found that postprandial inflammation is augmented in obese and overweight individuals as cytokines are secreted by adipose tissue [16,61]. The quantity of fat is thought to have greater impact on postprandial inflammatory response in overweight and obese than in healthy lean individuals [15]. This hypothesis is supported by a recent study which reported that the consumption of a high fat mixed meal increased plasma IL-6 and hsCRP concentrations compared with water (control) in 10 obese males [62] (Table 1). The mixed meal consisted of 57.5 g dietary fat (with 29 g saturated fat) derived from bacon, egg muffin, 2 hash browns with a glass of caramel-flavored milk drink. The mixed meal however did not alter plasma TNF-α and adiponectin concentrations and the similar observations were reported after the water intake. Manning et al. [16] however reported that both low and high fat meals increased plasma IL-6 concentrations but had no effect on plasma TNF-α and IL-8 concentrations in 15 obese women. The low fat meals were mashed potato (12 g fat) or all bran (13 g fat), whereas high fat meals were 200 g instant mashed potato enriched with cream (high SFA, 71 g), olive oil (high monounsaturated fats (MUFA), 72 g) or canola oil (high PUFA, 72 g). The low and high fat meals were not iso-caloric and the obese women ingested fats in proportion with their body weight. These conflicting findings may be explained by the differences in study design; (1) gender (men *vs.* women), (2) control used (water *vs.* 12 g fat), (3) amount of fat in high fat meals (57.5 g *vs.* 0.6 g/kg in proportion with body weight, which is equivalent to 72 g total fat), (4) composition of mixed meals and (5) measurement time point.

**Table 1:** Acute effects of the amount of dietary fats on inflammatory response in obese or overweight individuals

**Subjects: n (F/M)**	**Postprandial duration**	**Test meal**	**Inflammatory response in overweight/obese individuals**	**Remarks**	**References**
Lean: 10 (-/10) Obese: 10 (-/10) T2DM: 10 (-/10)	0, 1, 2, 3, 4, 5, 6 h	HFM: 57.5 g fat (29 g SFA) *A bacon and egg muffin, 2 hash browns, caramel flavored milk drink (250 ml of 4% fat milk, 4 teaspoons skim milk powder and 1 teaspoon caramel flavor)*	IL-6, hsCRP: HFM ↑ over time; NS TNF-α: =; NS Total adiponectin: HFM ↑ over time; NS HMW adiponectin: =; NS	Sample size was calculated based on the changes of adiponectin, but not other markers	[62]
		Water (control)		Small sample size	
Lean: 14 (14/-); obese: 15 (15/-)	0, 1, 4, 6 h	SFA: 71 g fat (18 g palmitate) *Instant potato with 16 g cream/kg body weight, 100 ml hot water*	IL-6: ↓ at 1 h, ↑ at 6 h; NS IL-8, TNF-α: =; NS	Timing of menstrual cycle and oral contraceptive usage were not taken into account	[16]
		MUFA: 72 g fat (9 g palmitate ) *Instant potato with 0.6 g/kg body weight of olive oil, 160 ml hot water*		Subjects with hypertension and dyslipidemia were included	
		n-6 PUFA:72 g fat (5 g palmitate) *Instant potato with 0.6 g/kg body weight of canola oil, 160 ml hot water*		Test meals were not iso-caloric Small sample size.	
		LFM–potato: 12 g fat (3 g palmitate) *Instant potato, 160 ml hot water.*			
		LFM – bran: 13 g fat (3 g palmitate) *All-Bran, 40 ml trim milk, and 2 cooked eggs*			
		*test meals for obese subjects only			
Obese: 38 (-/38)	0, 4, 8 h	60 g fat/m^2^ body surface *Eggs, cheese, toast, peanut 7, whipped cream, peaches and milk*	IL-6: ↑ at 6 h TNF-α: ↓ at 4 h	Control not included	[63]
Overweight: 15 (2/13)	0, 0.5, 1, 2, 4, 6, 8 h	82 g fat (36.9 g SFA) *Fried potatoes, fried eggs, Emmenthal cheese, Italian rose-shaped dinner rolls, with 500 ml of low sugar content beverage*	IL-6, TNF-α: ↑ over time	Small sample size Control not included Short postprandial period	[64]
Lean: 10 (5/5); Obese: 8 (5/3)	0, 1, 2, 3 h	HFM, 60 g fat *Big Mac, large French fries, a large Coke, and apple pie*	ROS generation and NF-κB binding : ↑ over time	Small sample size Short postprandial period Control not included	[15]

HFM, high fat meal; LFM, low fat meal; SFA, saturated fats; MUFA, monounsaturated fats; PUFA, polyunsaturated fats; % en, percentage energy; IL, interleukin; hsCRP, high-sensitivity C-reactive protein; TNF-α, tumor necrosis factor- alpha; sICAM-1, soluble intracellular adhesion molecule-1; sVCAM-1, soluble vascular adhesion molecule-1; HMW, high molecular weight; AUC, area under the curve; ROS, reactive oxygen species; NF-κB, nuclear factor kappa-β; ↓, reduced postprandial concentrations; ↑, increased postprandial concentrations; =, no postprandial/after meal effect; ↑↑, higher postprandial increment compared to other meals; NS, no significant difference between meals.

The discrepancies observed in these two studies may further be explained by a study conducted by Blackburn et al. [63]. A relatively larger sample size with 38 obese men ingested a high fat mixed meal consisted of eggs, cheese, toast, peanut butter, whipped cream, peaches and milk. The total fat was given at 60 g/m^2^ body surface area. This has resulted in an increase in plasma IL-6 concentrations but TNF-α and hsCRP concentrations remained unchanged. Miglio et al. [64] however observed an increase in postprandial plasma TNF-α concentrations following the ingestion of 82 g high fat mixed meal. In the study, 15 overweight men and women ingested a high fat mixed meal consisted of fried potatoes, fried eggs, Emmenthal cheese, and Italian rose-shaped dinner rolls, served with 500 ml low sugar content beverage. These discrepant of findings could be attributed to the different time point of blood sampling. The results observed by Blackburn et al. [63] at 4 h after meal commencement may have prevented the notice of an early increase in plasma TNF-α concentrations, as observed by Miglio et al. [64] at 1 h after meals. Amount of fat used by Miglio et al. [64] was higher compared to other studies [16,62,63], but a previous study reported that the ingestion of meals enriched with 50 g of fats enabled the detection of postprandial changes in plasma IL-6 and TNF-α concentrations in healthy subjects [65]. This suggests that the studies discussed have incorporated sufficient amount of fats to detect the postprandial changes in plasma cytokines.

The activation of NF-kB has been reported to regulate the release of cytokines during postprandial period [66]. Patel et al. [15] reported that a high fat, high carbohydrate meal increased the ROS generation and NF-κB binding activities in 8 obese subjects (3 men and 5 women). In the study, subjects consumed a high fat mixed meal consisted of a Big Mac, a large French fries, a large Coke and an apple pie, providing approximately 60 g of fats. However, the postprandial duration was relatively short (3 h) and changes in plasma cytokines concentrations were not measured; hence the relation between NF-κB and the release of cytokines deserves further investigation. Taken together the evidence available, the consumption of high fat meals may lead to the increase of postprandial inflammatory markers, in particular plasma IL-6, as well as the ROS generation and NF-κB binding activities in overweight and obese individuals.

### Type of dietary fats on postprandial inflammatory response

Masson and Mensink [67] reported that in 13 overweight men, plasma IL-6, TNF-α and soluble vascular adhesion molecule-1 (sVCAM-1) concentrations decreased after n-6 PUFA meal, while the markers were increased after SFA meal (Table 2). The subjects were randomly assigned to high fat meal enriched with 50 g butter (SFA meal), or 40 g margarine plus 10 g safflower oil (n-6 PUFA meal). Both test meals were served in the form of muffins (two per subject) with a glass of water (250 ml). Interestingly, decreased plasma MCP-1 concentrations were observed after both high fat meals, while no meal effect was reported for plasma IL-8 and soluble intracellular adhesion molecule-1 (sICAM-1) concentrations. In contrast, Manning et al. [16] observed that all high fat meals regardless of type of fats increased plasma IL-6 concentrations in a similar manner but had no impact on plasma IL-8 and TNF-α concentrations in 15 obese women. The inconsistencies of results reported by these two studies may be explained by the gender differences (men *vs*. women) of the subjects as discussed earlier. Furthermore, the differing content of linoleic acids (20 g *vs.* 14 g for n-6 PUFA meals), may attribute to the discrepant findings. Evidence from *in vitro* findings suggested a pro-inflammatory effect of n-6 PUFA, however the effect is uncertain in human studies [68]. In addition, the sources of SFA (butter *vs*. cream) used may attribute to the diversifying results. Butter contains substantial amount of medium chain triglycerides which may be better absorbed compared with cream with different physical characteristics [69].

**Table 2:** Acute effects of the type of dietary fats on inflammatory response in obese or overweight individuals

**Subjects: n (F/M)**	**Postprandial duration**	**Test meal**	**Inflammatory response in overweight/obese individuals**	**Remarks**	**References**
Overweight: 13 (-/13)	0, 15, 30, 45, 60, 90 min, 2, 3, 4, 5, 6, 7, 8 h	SFA: 56.6 g fat (33.9 g SFA) *2 muffins with butter and 250 ml water*	IL-6, TNF-α, sVCAM-1: SFA ↑, PUFA ↓ MCP-1: ↓ over time; NS sICAM-1: = ; NS	Small sample size	[67]
		n-6 PUFA: 60.5 g fat (12.9 g SFA, 21.8 g PUFA) *2 muffins with 40 g butter and 10 g margarine, and 250 ml water*			
		*A low fat lunch was served after 3 h*			
Lean: 14 (14/-) Obese: 15 (15/-)	0, 1, 4, 6 h	SFA: 71 g fat (18 g palmitate) *Instant potato with 16 g cream/kg body weight, 100 ml hot water*	IL-6: ↓ at 1 h, ↑ at 6 h; NS IL-8, TNF-α: =; NS	Timing of menstrual cycle and oral contraceptive usage were not taken into account	[16]
		*MUFA: 72 g fat (9 g palmitate)* *Instant potato with 0.6 g/kg body weight of olive oil, 160 ml hot water*		Subjects with hypertension and dyslipidemia were included	
		n-6 PUFA:72 g fat (5 g palmitate ) *Instant potato with 0.6 g/kg body weight of canola oil, 160 ml hot water*		Test meals were not iso-caloric Small sample size	
		LFM–potato: 12 g fat (3 g palmitate) *Instant potato, 160 ml hot water.*			
		LFM – bran: 13 g fat (3 g palmitate) *All-Bran, 40 ml trim milk, and 2 cooked eggs*			
		*test meals for obese subjects only			
Lean: 18 (-/18) Obese: 18 (-/18)	0, 2, 4 h	SFA: 95 g fat (51 g SFA) *Shakes containing low fat yogurt, low fat milk, strawberry flavor, sugar and 95 g palm oil*	IL-8: ↑ over time; NS IL-6: ↓ over time; NS CRP: =; NS	Short postprandial period Small sample size	[61]
		MUFA: 95 g fat (8 g SFA) *Shakes containing low fat yogurt, low fat milk, strawberry flavor, sugar and 95 g high oleic sunflower oil*			
		n-3 PUFA: 95 g fat (32 g SFA) *Shakes containing low fat yogurt, low fat milk, strawberry flavor, sugar and 40 g palm oil + 55 g Marinol D40 (40% DHA).*			
Lean: 18 (-/18) Obese: 18 (-/18) Obese diabetic: 6 (-/6)	0, 2, 4 h	SFA: 95 g fat (51 g SFA) *Shakes containing low fat yogurt, low fat milk, strawberry flavor, sugar and 95 g palm oil*	IL-1β and TNF-α: =; NS PBMC MCP-1 and IL-8: MUFA and n-3 PUFA ↑↑, SFA ↑ at 4 h	Short postprandial period Small sample size	[70]
		MUFA: 95 g fat (8 g SFA) *Shakes containing low fat yogurt, low fat milk, strawberry flavor, sugar and 95 g high oleic sunflower oil*			
		n-3 PUFA: 95 g fat (32 g SFA) *Shakes containing low fat yogurt, low fat milk, strawberry flavor, sugar and 40 g palm oil + 55 g Marinol D40 (40% DHA)*			
Obese/overweight: 10 (6/4)	0, 1, 2, 4, 6 h	SFA: 83 g fat *Refined palm oil blended with 1% milk, strawberry flavored syrup, low fat frozen, and non-fat dry milk powder.*	CRP: ↑; over time NS TNF-α: ↓ over time; NS NF-κB AUC (4 h): PUFA ↑↑, SFA↑ VCAM-1: =; NS ICAM-1: =; MUFA ↓↓ *vs.* SFA, n-3 PUFA	Small sample size Test meals were not iso-caloric	[71]
		MUFA: 83 g fat *Refined olive oil blended with 1% milk, strawberry flavored syrup, low fat frozen, and non-fat dry milk powder.*			
		n-3 PUFA: 85 g fat *Refined olive oil + 4 g n-3FA from 8 g fish oil supplement pills (300 mg EPA, 200 mg DHA/g), blended with 1% milk, strawberry flavored syrup, low fat frozen, and non-fat dry milk powder.*			

LFM, low fat meal; SFA, saturated fats; MUFA, monounsaturated fats; PUFA, polyunsaturated fats; % en, percentage energy; IL, interleukin; hsCRP, high-sensitivity C-reactive protein; TNF-α, tumor necrosis factor- alpha; sICAM-1, soluble intracellular adhesion molecule-1; sVCAM-1, soluble vascular adhesion molecule-1; TAG, triacylglycerols; MCP-1, monochemoattractant protein-1; PBMC, peripheral blood mononuclear cells AUC, area under the curve; ↓, reduced postprandial concentrations; ↑, increased postprandial concentrations; =, no postprandial/after meal effect; ↑↑, higher postprandial increment compared to other meals; NS, no significant difference between meals.

The following three studies reported that n-3 PUFA had no favorable impact on the postprandial responses of plasma cytokines when compared with MUFA or SFA rich oils. Esser et al. [61] provided 18 obese men SFA (95 g palm oil), MUFA (95 g high oleic sunflower oil), or n-3 PUFA (40 g palm oil plus 55 g Marinol D-40) enriched shakes. The study reported that the high fat meals increased plasma IL-8 concentrations, and reduced plasma IL-6 concentrations in a similar magnitude but no impact on plasma CRP concentrations. Another similar study by Van Dijk et al. [70] reported null effects on plasma IL-1β and TNF-α concentrations in obese men, irrespective of the type of fats consumed (refined palm oil as SFA, refined olive oil as MUFA, and refined olive oil plus 4 g of n-3FA from 8 g fish oil supplement pills as n-3PUFA). The study however, observed lower peripheral blood mononuclear cells (PBMC) MCP-1 and IL-8 concentrations after SFA compared with MUFA and n-3 PUFA high fat meals in obese men. This observation concurs with an earlier study in 10 obese overweight subjects [71]. Although the postprandial effect on plasma cytokines (CRP, TNF-α, sICAM-1 and sVCAM-1) did not vary, n-3 PUFA was found to activate NF-κB expression compared with SFA. The authors explained that the lower activation of NF-κB by SFA may be attributed to the palmitate content in the palm oil, as *in vitro* studies have shown that high levels of palmitate may suppress NF-κB activation [27]. Despite the diminished effect on plasma cytokines, the activation of NF-κB may suggest that the synthesis and secretion processes of plasma cytokines may not occur concurrently in the cell and extracellular tissue, hence suggesting that a complex pathophysiological mechanism involved.

Evidence to-date suggests that there is no clear beneficial effect of the consumption of PUFA rich meals on postprandial plasma inflammatory cytokines. On the other hand, SFA rich meals may not be detrimental in an acute feeding scenario in obese and overweight individuals. However, high fat meal may promote elevated postprandial inflammatory response, with limited evidence on varying cytokines. It is also important to understand that the repetitive exposure of dietary fatty acids on the whole body homeostasis may only be observed in long-term dietary intervention studies.

## Chronic dietary intervention

### Effects of the type of dietary fats on inflammation

n-6 PUFA (particularly of linoleic acid), is negatively viewed as pro-inflammatory considering the fact that it can be metabolized into AA and subsequent undesirable metabolites. Furthermore, n-6 PUFA may compete for cyclooxygenase thus reducing the formation of anti-inflammatory mediators from n-3 PUFA [72]. Nevertheless, a rigorously well conducted systematic review reported that no clinical evidence to-date were able to suggest the pro-inflammatory effects of linoleic acid in healthy, noninfant individuals [73]. However these findings may need further confirmation in overweight and obese individuals. The baseline levels of inflammatory markers in lean healthy individuals are appreciably lower compared to an overweight population. Hence the impact of dietary fatty acids may virtually be negligible in lean healthy population. Two recent studies in obese individuals however have reported discrepant findings (Table 3). Kralova Lesna et al. [74] conducted a 3-week dietary intervention with predominant PUFA (25% en PUFA from vegetable oils) or SFA (29% en SFA from animal fat content) in 14 overweight, dyslipidemic and postmenopausal women. The study reported that a relatively high intake of PUFA decreased plasma CRP compared with baseline but no comparison was made between diets. The small sample size and short study duration with limited statistical interpretation may reduce the strength of findings. In addition, Bjermo et al. (2012) reported that n-6 PUFA decreased plasma IL-1RA and TNF-R2 concentrations compared with a SFA-enriched diet after a 10-week iso-caloric dietary intervention in 61 abdominally obese individuals [75]. n-6 PUFA (from sunflower oil) was exchanged at 10% en with SFA derived predominantly from butter. The well powered study with longer duration may explain the positive findings of n-6 PUFA. However, the results generated may be less convincing based on the high noncompliance reported (PUFA, 16%; SFA, 34%). Furthermore, it is to be noted that the non-equally distributed participants (16-31%) on antihypertensive and lipid-lowering drugs, may confound the levels of circulating cytokines in a parallel study [76,77].

**Table 3:** Chronic effects of the type of dietary fats on low grade inflammation in obese or overweight individuals

**Subjects: n (F/M)**	**Design**	**Dietary intervention**	**Inflammatory response**	**Remarks**	**References**
Overweight/obese: 14 (14/-)	Crossover; 3-week, 1 week wash-out	PUFA 40% en total fat (25% en PUFA, 8.5% en SFA) *Fat from vegetable sources*	CRP: PUFA↓ *vs baseline* IL-18: NS vs baseline	Small sample size Dyslipidemic and postmenopausal women were recruited	[74]
		SFA 42% en total fat (29% en SFA, 3% en PUFA) *Dairy and animal fats*	*No comparison was made between diets	Short dietary intervention	
		*Iso-caloric diet (2738 kcal) with ~21% en exchange between PUFA and SFA*			
Abdominally overweight: 61 (gender not specified) n-6 PUFA:32 SFA: 29	Parallel; 10-week	n-6 PUFA 40% en total fat (10% en SFA, 13.5% en LA) *Scones (baked using sunflower oil), margarine, sunflower oil and sunflower seeds*	IL-1RA, TNF-R2: n-6 PUFA↓ *vs* SFA CRP, IL-6, IL-1β and IL-10: NS	Subjects used antihypertensive and lipid lowering drugs.	[75]
		SFA 40% en total fat (20% en SFA, 4% en LA) *Scones (baked using butter), butter and butter*		Low compliance: n-6 PUFA diet (n = 27); SFA diet (n = 19)	
		*Iso-caloric diet (2000 kcal) consisted with 10% en exchange between n-6 PUFA and SFA. Key fat sources were provided*			
Overweight and obese: 76 (63/13) Krill: 25 (22/3) Menhaden: 26 (21/5) Control:25 (20/5)	Parallel; 4-week	Krill oil: 90 mg DHA + 216 mg EPA Menhaden oil: 178 mg DHA + 212 mg EPA Control: 2 g olive oil *4 x 500 mg capsules/day for each supplementation*	hsCRP: NS	Habitual diet was not controlled	[78]
Sedentary overweight: 138 (93/45)	Parallel: 4-month	n-3 PUFA: 2.5 g/day *Fish oil (6 x 500 mg capsules/d);EPA:DHA ratio is 7:1*	TNF-α, IL-6: both doses ↓ *vs* placebo; NS	Calorie and fatty acid composition of habitual diets were not standardized	[79]
n-3 PUFA 2.5 g/day: 46 (29/17) n-3 PUFA: 1.25 g/day: 46 (28/18) Placebo: 46 (36/10)		n-3 PUFA: 1.25 g/day Placebo: 3 g *Mixture of palm, olive, soy, canola and cocoa butter oils; (SFA:MUFA:PUFA ratio = 37:42:21)*			
Severely obese:55 (46/9) n-3 PUFA: 27 (23/4) Control: 28 (23/5)	Parallel: 8-week	n-3 PUFA: 4 x 1 g capsules/day (3.36 g EPA + DHA) Control: 5 g butterfat	IL-6: n-3 PUFA ↓ hsCRP: NS	30 subjects used supplementary medication such as antihypertension and proton pump inhibitors Incomplete dietary records	[82]
		*Iso-caloric diet consisted of 30% en fat, 15% protein and 55% en carbohydrate*	SAT gene expression of CCL2, CCL3, H1F1A and TGFB1: n-3 PUFA ↓		
			EPA- and DHA-derived eicosanoids synthesis in SAT and VAT: n-3 PUFA ↑		
Abdominally overweight/ obese: 51 (40/11)	Parallel: 8-week	ALA: 11 g/day flaxseed oil Control: habitual diet	IL-6, TNF-α, CRP: NS		[83]
ALA: 27 (21/6) Control: 24 (19/5)		*Iso-caloric diet (~2000 kcal) with a balance of SFA and MUFA intake*			

% en, percentage energy; IL-1RA, interleukin-1 receptor antagonist; TNF-R2, tumor necrosis factor- receptor 2; hsCRP, high-sensitivity C-reactive protein; IL, interleukin; TNF-α, tumor necrosis factor- alpha; ALA, alpha linolenic acid; SFA, saturated fats; MUFA, monounsaturated fats; PUFA, polyunsaturated fats; DHA, docosahexaenoic acid; EPA, eicosapentaenoic acid; VAT, visceral adipose tissue; SAT, subcutaneous adipose tissue; CCL, chemokine (C-C) motif ligand; HIF1A, hypoxia-inducible factor 1-alpha; TGFB1, transforming growth factor β1; NS, no significant difference between diets; ↓, reduced concentrations; ↑, increased concentrations.

The anti-inflammatory effects of n-3 PUFA has been confirmed in prospective epidemiological and *in vitro* findings. Thus, the anti-inflammatory potential of n-3 PUFA has been put to a rigorous test in clinical trials. However, five studies reported conflicting findings. Maki et al. [78]reported that supplementation of krill oil (216 mg EPA, 90 mg DHA), menhaden oil (212 mg DHA, 178 mg EPA) or control (2 g olive oil) for 4-week to 25 overweight individuals did not alter plasma CRP. In contrary, Kiecolt-Glaser et al. (2012) reported that the consumption of two doses of n-3 PUFA (1.25 *vs* 2.5 g/day, EPA: DHA ratio is 7:1) for 4 months in 46 abdominally overweight individuals resulted in discernable reduction in plasma TNF-α and IL-6 concentrations [79]. It is plausible that the longer duration with sufficiently powered design enable the detection of positive findings. Furthermore, ideal balance of EPA to DHA may play a critical role as evidence suggests that EPA may be more anti-inflammatory than DHA [80,81]. In agreement with Kiecolt-Glaser et al. [79], a recent study found that 55 severely obese nondiabetic patients provided with 3.36 g n-3 PUFA (EPA, DHA) for 8-week experienced a marked decrease in plasma IL-6 but not hsCRP concentrations [82]. Interestingly, the study also reported that n-3 PUFA decreased the expression of varying inflammatory genes in subcutaneous adipose tissue and increased the production of eicosanoids in both visceral and subcutaneous adipose tissue compared with butter fat (control). The beneficial effect of n-3 PUFA was evident in severely obese patients (BMI ≥ 40 kg/m^2^) and this again proven that the impact of dietary fats on plasma cytokines is attributed to the baseline levels of circulating cytokines.

To investigate the plant-based n-3 PUFA, namely ALA, Nelson et al. [83] fed healthy abdominally overweight individuals with flaxseed oil (5% en ALA of total energy intake or 11 g/day, n = 27) or habitual diet (n = 24), in a parallel design for 8-week. The targeted fat exchange with ALA was achieved at 10 g/day or 4.6% en of total fat intake as reported by self-reporting dietary record. No significant changes was observed for plasma IL-6, TNF-α and CRP concentrations. However, measurable increase of EPA and DHA was detected in erythrocyte cell membranes suggesting the conversion of ALA to EPA and DHA in the relatively short study duration. The primary differences between this study and previously reported were the fat source (plant-based ALA), younger age (~ 38 y), and relatively healthy overweight individuals. This further supports the hypothesis that the baseline levels of cytokines were too low for any discernable changes. Taken together, the effect of dietary fats, in particular n-6 PUFA required further confirmation. The beneficial effects of n-3 PUFA may only be observed in severely obese individuals with higher baseline levels of plasma cytokines. Further studies bridging the gap of information are needed.

### Restricted calorie diets with varying amount of dietary fats on inflammation

Weight loss lowers the risk for developing cardiovascular diseases, but sustained weight loss is difficult to achieve. In this context, dietary approach is needed. The effect of restricted diets with varying amount of dietary fats on low grade inflammation is uncertain. Sharman and Volek [84] conducted a 6-week crossover dietary intervention with reduced energy diets (low fat *vs* very low carbohydrate (VLCKD), ~1500 kcal) in 15 overweight men (Table 4). A low level of ketosis was ascertained as an indication of compliance. Plasma TNF-α, IL-6, hsCRP and sICAM-1 concentrations did not differ between diets but appreciably reduced compared with baseline. Forsythe et al. [85] conducted a parallel study with longer period dietary intervention (12-week) in a group of overweight individuals with dyslipidemia. Similar weight reduction was achieved in a longer period of time as compared with Sharman and Voleks [84]. The study reported that both diets significantly decreased concentrations of several pro-inflammatory cytokines but profound alterations were observed after VLCKD intervention as evident by remarkable decrease in an array of markers (TNF-α, IL-6, IL-8, MCP-1, E-selectin, sICAM and plasminogen activator inhibitor-1 (PAI-1)). The authors suggested that the lower inflammatory response in VLCKD may be correlated with the increased plasma n-6 PUFA levels attributed by a higher saturated fat intake. However, the higher weight loss achieved by VLCKD group may also contribute to a more remarkable improvement in inflammatory status.

**Table 4:** Chronic effects of restricted calorie diets with varying amount of fats on low grade inflammation in obese and overweight individuals

**Subjects: n (F/M)**	**Design**	**Dietary intervention**	**Inflammatory response**	**Remarks**	**References**
Overweight: 15 (-/15)	Crossover: 6-week	VLCKD 60% en total fat, 10% en CHO, 30% en protein Unlimited type of fat or cholesterol amount from sources such as beef, poultry, fish, oils, various nuts/seeds, moderate amount of vegetables, salads with low-carbohydrate dressings, moderate amount of cheese, eggs, protein powder, water/ low-carbohydrate diet drinks, low-carbohydrate bars and shakes *Customied diabetic exchange lists were used.	TNF-α, IL-6, hsCRP, sICAM-1: both VLCKD & LFD ↓ *vs* baseline: NS sP-sel: NS *Body weight: VLCKD ↓ 6.5 kg, LFD ↓3.7 kg*	Protein intake not standardised across diets	[84]
		LFD 25% en fat, 55% en CHO, 20% en protein (< 10% SFA, < 300 mg cholesterol)			
Overweight: 20	Parallel: 12-week	VLCKD 60% en fat 10% en CHO 30% en protein *Unlimited amount of beef, poultry, fish, eggs, oils and heavy creams; moderate amount of hard cheeses, low-carbohydrate vegetables and salad dressings; small amount of nuts, nut butters and seeds.*	TNF-α, IL-8, MCP-1, E-selectin, sICAM-1 PAI-1: both VLCKD & LFD ↓ *vs* baseline; VLKCD ↓↓ IL-6, CRP, VEGF, P-sel, EGF and sVCAM-1: both VLCKD & LFD ↓ *vs* baseline; NS	Protein intake not standardised across diets Dyslipidemic subjects were included	[85]
		LFD 25% en fat, 55% en CHO, 20% en protein (< 10% SFA, < 300 mg cholesterol) Whole grains (bread, cereals and pastas), fruit/fruit juices, vegetables, vegetables oils, low at dairy, and lean meat products *Standard diabetic exchange lists were used.	*Body weight: VLCKD ↓ 5.6 kg, LFD ↓3.7 kg*		

VLCKD, very low carbohydrate ketogenic diet; LFD, low fat diet; CHO, carbohydrate; % en, percentage energy; SFA, saturated fats; TNF-α, tumor necrosis factor- alpha; IL, interleukin; hsCRP, high-sensitivity C-reactive protein; sICAM-1, soluble intracellular adhesion molecule-1; sP-sel, soluble P-selectin; MCP-1, monochemoattractant protein-1; PAI-1, plasminogen activator inhibitor-1; VEGF, vascular endothelial growth factor; EGF, epidermal growth factor; sVCAM-1,soluble vascular adhesion molecule-1; NS, no significant difference between diets; ↓, reduced concentrations; ↓↓, greater reduction compared to the other diets.

### Restricted calorie diets with varying type of dietary fats on inflammation

In recent years, the effect of n-3 PUFA on weight loss has been studied extensively. Four studies have been identified in this respect (Table 5). Munro and Garg [86] reported that a low energy diet combined with n-3 PUFA (8% en of total fat intake, 0.42 g EPA + 1.62 g DHA from fish oil) or control (MUFA, sunola oil) for 12-week did not differ on plasma adiponectin, leptin, hsCRP and IL-6 concentrations. A significant weight loss was achieved and a 2-fold increase in plasma EPA and DHA suggested compliance. Nevertheless, Kratz et al. [87] provided 16 women and 10 men a diet rich in n-3 PUFA (3.5% en of total fat intake, from both plant and marine sources) or a control diet (3.2% en intake from MUFA) for a consecutive 2-week lead-in followed by a 2-week iso-caloric period and a 12-week ad libitum period. Weight loss resulted in an expected increase in adiponectin levels during the ad libitum period. Both n-3 PUFA and control diets however did not alter plasma adiponectin levels. The authors discussed that the dose of fish oil provided (725 mg) is equivalent to the amount of 125-250 g fatty fish/day, which is on the higher end of human consumption.

**Table 5:** Chronic effects of restricted calorie diets with varying type of fats on low grade inflammation in obese and overweight individuals

**Subjects: n (F/M)**	**Design**	**Dietary intervention**	**Inflammatory response**	**Remarks**	**References**
Overweight: 33 (22/11)	Parallel: 12-week	n-3 PUFA: *6 capsules of 1 g fish oil daily (0.42 g EPA + 1.62 g DHA/d)*	Body weight: n-3 PUFA ↓4.2 kg, placebo ↓ 3.17 kg; NS		[86]
n-3 PUFA:15 (10/5) Placebo:18 (12/6)		Placebo (MUFA): *6 capsules of 1 gunola oil daily*	Leptin, adiponectin, hsCRP, IL-6: NS		
		*Daily intake were 5000 kJ for female and 6000 kJ for males with ~30% en total fat intake*	TNF-α: n-3 PUFA ↓ *vs* baseline; NS		
Overweight: 26 (16/10)	Parallel: 14-week	**Phase 1 (lead-in):** 34.3% en fat (4.8% en n-6, 0.5% en n-3 PUFA)	Body weight after Phase 3: Both diets ↓ (~3.5% body weight); NS		[87]
n-3 PUFA: 13 (8/5) control: 13 (8/5)	*Phase 1: 2-week* *Phase 2: 2-week* *Phase 3:12-week*	*Both n-3 PUFA and control groups consumed the same diet* **Phase 2 (iso-caloric, 1800 kcal)):** n-3 PUFA: 12 capsules of 725 mg fish oil (2.88 g n-3 PUFA) 34.7% en fat (n-6 PUFA, 4.9% en, n-3 PUFA, 3.6% en) *Main source of n-3 PUFAs: canola and flaxseed oils, and margarines rich in ALA*	Adiponectin: both diets ↑ *vs* baseline; NS HMW adiponectin: NS		
		Control: high oleic sunflower oil capsules 34.3% en total fat (4.8% en n-6, 0.5% en n-3 PUFA) *Main source of oils: high oleic safflower and sunflower oils, and margarines based on these oils*			
		**Phase 3 (ad libitum):** 115% of the amount of food provided in phase 1 and 2			
Obese: 11 (-/11) Phase 1:11(-/11) Phase 2:8 (-/8)	Cross-over Phase 1: 6-week Phase 2: 8-week *(very low calorie diet, 4-week; restricted energy diet, 2-week; weight sustained diet, 2-week)*	**Phase 1 (fish oil study):** n-3 PUFA: 0.6 g EPA + 0.5 g DHA Control: 6 capsules 500 mg of high oleic sunflower oil *with ad-libitum Dutch diet* *No fish consumption is allowed*	Postprandial IL-6, CRP, sTNF-R55, sTNF-R75, PAI-1 antigen: weight loss ↓: NS TNF-α: NS	Small sample size	[5]
		**Phase 2 (weight loss period):** Very low calorie diet: 480 kcal/day with s*hakes + 250 g vegetables and fruits except banana*			
	*Postprandial challenges at the end of each intervention: 0, 2, 4 h*	Restricted energy diet: 1000 kcal/day with m*ixed solid energy-restricted diet with a recommended composition*			
		Weight sustained diet: *Diet at commensurate calories to maintain the newly achieved weights* Postprandial meals: 50.1 g fat, milkshake			
Obese: 11 (-/11)	Cross-over	**Phase 1 (fish oil study):** n-3 PUFA: 0.6 g EPA + 0.5 g DHA	Fasting sICAM-1, hsCRP: weight loss ↓; NS sE-sel, MCP-1: NS	Small sample size	[6]
Phase 1:11(-/11) Phase 2:8 (-/8)	Phase 1: 6-week Phase 2: 8-week *(very low calorie diet, 4-week; restricted energy diet, 2-week; weight sustained diet, 2-week)*	Control: 6 capsules 500 mg of high oleic sunflower oil *with ad-libitum Dutch diet* *No fish consumption is allowed*	Postprandial sICAM, hsCRP: weight loss ↓ MCP1: PUFA & weight loss ↓ sel: NS		
		**Phase 2 (weight loss period):** Very low calorie diet: 480 kcal/day with s*hakes + 250 g vegetables and fruits except banana*			
	*Postprandial challenges at the end of each intervention: 0, 2, 4 h*	Restricted energy diet: 1000 kcal/day with m*ixed solid energy-restricted diet with a recommended composition* Weight sustained diet: *Diet at commensurate calories to maintain the newly achieved weights*			
		Postprandial meals: 50.1g fat, milkshake			

% en, percentage energy; DHA, docosahexaenoic acid; EPA, eicosapentaenoic acid; HMW, high molecular weight; MUFA, monounsaturated fats; PUFA, polyunsaturated fats; hsCRP, high-sensitivity C-reactive protein; IL, interleukin; PAI-1, plasminogen activator inhibitor-1; sTNF-R55, soluble tumor necrosis factor receptor 55; sTNF-R75, soluble tumor necrosis factor receptor 75; sICAM-1, soluble intracellular adhesion molecule-1; sE-sel, soluble E-selectin; MCP-1, monochemoattractant protein-1; NS, no significant difference between diets; ↓, reduced concentrations; ↑, increased concentrations.

Two similar studies were conducted to compare the effects of n-3 PUFA (1.1 g/day n-3 PUFA with 0.6 g EPA, 0.5 g DHA) and MUFA (control, high oleic sunflower oil) supplementation *vs* weight loss on inflammatory biomarkers in 11 healthy moderately obese men [5,6]. The subjects consumed a succession of very low calorie diet for 4-week, followed by a 2-week restricted calorie diet and a 2-week weight sustained diet. At the end of dietary intervention, fasting blood samples were collected followed by a 4 h postprandial high fat challenge (50 g fat with 2:2:1 ratio of SFA:MUFA:PUFA). Weight loss (~9.4 kg) reduced both fasting and postprandial sICAM, hsCRP and postprandial IL-6, sTNF-R55, sTNF-R75 and PAI-1 antigen. MCP-1 was lowered by both n-3 PUFA and weight loss. Hence, weight loss rather than n-3 PUFA supplementation had prominent impact on low grade inflammation.

## Strengths and limitations

The current evidence provides insight on the influence of amount and type of dietary fats on inflammatory response in both acute and chronic dietary intervention studies in obese and overweight individuals. The present review is not a systematic review with grading tools applied, however the limited evidence in hand suggests that dietary fats may play a pivotal role in modulating low grade inflammation in a potentially vulnerable group of population to disease progression. However, the diverting information generated so far highlights the need for better designed dietary intervention in order to unravel the crucial role of fatty acids on low grade inflammation. Most of the studies reported are underpowered hence reflecting variations which may mask the true effects of dietary treatment. In addition, the comparisons made were based on dietary fats as a whole rather than a head-to-head comparison of individual fatty acid per se. The results generated thus may not be extrapolated to individual fatty acid. In addition, the source of dietary fats whether from animal or vegetable origin may generate remarkable varying findings. This is in particular obvious when palm oil was used as SFA instead of butter or cream as discussed. The physical characteristics such as solid fat content or melting point have been reported to produce discernable outcome on postprandial lipemia [88]. Furthermore, population difference is another concern to be noted. The subjects recruited ranging from healthy overweight to severely obese with mildly elevated dyslipidemia, young to elder women to men may generate differing outcome. For acute studies in particular, the minimum amount of fat required to induce a remarkable postprandial inflammatory response is 50 g [65]. The composition of test meals i.e. mixed meal *vs* milk shake may generate differing outcome which may complicate data interpretation. The selection of control meal may also affect the results generated. Furthermore, proper blood sampling time point and procedure may greatly influence the detection of postprandial changes. Collective information has suggested that lower plasma IL-6 levels were observed at 1 hour after high fat meals, the missing time point may lead to different outcome [63,64]. Furthermore, diverting observation was obtained when different methods of blood sampling were used. It was reported that cannula rather than indwelling catheter causes minimal changes in plasma IL-6 [89]. However this may not be practical for study involving multiple blood samplings. The existing data on chronic studies highlights the need for proper designed studies. Most of the previous studies reported information on the intake of dietary fat using self-reporting dietary records. The major pitfall of dietary recall is the underestimation of dietary intake and lack of validation of methodology used [90]. In addition, the duration of dietary intervention in order to detect significant changes and dosage applied may also lead to inconsistency of findings. Taken together, future studies addressing these issues may provide more reliable and conclusive evidence on the role of dietary fatty acids on low grade inflammation.

## Conclusion

High fat meal may provoke inflammatory response in a postprandial state however the effect of type of dietary fats remained uncertain. Dietary fats, in particular n-3 PUFA may play a pivotal role in improving inflammatory status in severely obese individuals, however the impact may be marginal compared with weight loss. No conclusive evidence on the effect of type of dietary fatty acids on varying cytokines. A complete understanding on the role of dietary fatty acids on low grade inflammation merits further investigation. Information is needed based on rigorously well designed clinical studies.

## Abbreviations

SFA: Saturated fats; PUFA: Polyunsaturated fats; DHA: Docosahexaenoic acid; EPA: Eicosapentaenoic acid; TAG: Triacylglycerols; TNF-α: Tumor necrosis factor- alpha; IL-6: Interleukin-6; MCP-1: Monochemoattractant protein-1; CCR2: CC chemokine receptor 2; IL-10: Interleukin-10; IL-1Ra: Interleukin-1 receptor antagonist; JNK: Jun *N*-terminal kinase; NF-κB: Nuclear factor-kappa B; PBMC: Peripheral blood mononuclear cells; TLR: Toll-like receptor; ROS: Reactive oxygen species; LA: Linoleic acid; AA: Arachidonic acid; PGE2: Prostaglandin E2; ALA: Alpha linolenic acid; hsCRP: High-sensitivity C-reactive protein; sICAM-1: Soluble intracellular adhesion molecule-1; sVCAM-1: Soluble vascular adhesion molecule-1; MUFA: Monounsaturated fats; PAI-1: Plasminogen activator inhibitor-1.

## Competing interests

TKT and KN are providing consulting services to the Malaysian Palm Oil Board (MPOB). No potential conflict of interest was reported by CCY and CLF.

## Authors’ contributions

TKT, CCY and CLF contributed to the conception and design of the review as well as drafting. CCY, TKT and CLF performed the literature search. TKT and KN revised the manuscript. All authors read and approved the final manuscript.

## Authors’ information

TKT is a PhD graduate, and research officer at the Malaysian Palm Oil Board (MPOB). CLF is a postgraduate PhD student and CCY is a postgraduate master student, at University of Malaya. KN is a PhD graduate, and serves as a minister at the Embassy of Malaysia and Mission of Malaysia to the European Union.
